# The Growth of Soybean (*Glycine max*) Under Salt Stress Is Modulated in Simulated Microgravity Conditions

**DOI:** 10.3390/cells14070541

**Published:** 2025-04-03

**Authors:** Setsuko Komatsu, Haruka Misaki, Wei Zhu, Hisateru Yamaguchi, Keisuke Hitachi, Kunihiro Tsuchida, Atsushi Higashitani

**Affiliations:** 1 Faculty of Environment and Information Sciences, Fukui University of Technology, Fukui 910-8505, Japan; 2 Hangzhou Institute of Medicine, Chinese Academy of Sciences, Hangzhou 310018, China; zhuwei@him.cas.cn; 3 Department of Medical Technology, Yokkaichi Nursing and Medical Care University, Yokkaichi 512-8045, Japan; h-yamaguchi@y-nm.ac.jp; 4 Center for Medical Science, Fujita Health University, Toyoake 470-1192, Japantsuchida@fujita-hu.ac.jp (K.T.); 5 Graduate School of Life Sciences, Tohoku University, Sendai 980-8577, Japan

**Keywords:** proteomics, soybean, salt, simulated microgravity

## Abstract

The role of a simulated microgravity environment on soybean growth was investigated. The root grew more under simulated microgravity conditions than in the presence of gravity. However, root shortening due to salt stress did not occur in simulated microgravity conditions. To reveal these mechanisms by simulated microgravity environment on soybean root, a proteomic analysis was conducted. Proteomic analysis revealed that among 1547 proteins, the abundances of proteins related to phytohormone, oxidative stress, ubiquitin/proteasome system, cell organization, and cell wall organization were altered under stimulated microgravity compared with gravity. Membrane-localized proteins and redox-related proteins were inversely correlated in protein numbers due to salt stress under gravity and the simulated microgravity condition. Proteins identified by proteomics were validated for protein accumulation by immunoblot analysis. Superoxide dismutase and ascorbate peroxidases, which are reactive oxygen species-scavenging proteins, increased in soybean root under salt stress but not in the simulated microgravity conditions even under stress. The accumulation of 45 kDa aquaporin and 70 kDa calnexin in soybean root under salt stress were increased in the simulated microgravity conditions compared to gravity. These findings suggest that soybean growth under salt stress may be regulated through improved water permeability, mitigation of reactive oxygen species production, and restoration of protein folding under simulated microgravity conditions.

## 1. Introduction

Plants have developed numerous response mechanisms for adapting to a variety of stressful growth conditions, including salinity, drought, and extreme temperature [1]. Among abiotic stresses, salinity is one of the most frequently occurring yet least resolved environmental stresses, significantly reducing agricultural productivity [2]. Salt-tolerant plants have evolved many strategies to survive salt stress, including adjusting cellular osmolarity through the biosynthesis of osmoprotectants [3], maintaining cellular redox balance [4], and secreting salts outside the plant body from trichomes or glands [5]. Salt stress disrupts the root ion-transport system and inhibits nutrient absorption. Under salt stress, Na ions compete with K ions for uptake into the roots [6,7]. Extreme accumulation of Na ions in the cytoplasm not only interferes with the normal function of cell but also impedes absorption and transport of other essential ions [8,9]. These phenomena under salt stress lead to suppression of plant growth and an increase in wilting, ultimately leading to plant death.

Roots are continually sensing the surrounding environmental conditions, including light, water level, or salinity [10]. The root system architecture defines root morphology and structure and plays a key role in plant productivity by allowing efficient access to important soil resources [11,12]. Plant water and nutrient uptake is regulated by root system architecture [13]. A complex interplay of phytohormones controls the dynamic organization of crop root architecture. Among the various phytohormones, auxin acts as an endogenous regulator of root development in soil, from the early organogenesis to the formation of root hair via multiple signaling mechanisms [14]. The gene-expression regulatory networks controlled by heat-stress transcription factors regulate gravitropic responses via transcriptional control of heat-shock proteins, which are involved in regulating auxin transport in rice roots [15]. The relationship between directional gravitropism in roots and auxin is classified into four processes as follows: (i) gravity perception in gravity-sensing columella cells [16], (ii) gravity signaling after directional gravistimulation via LAZY1-like proteins [17], (iii) redistribution of auxin due to differences in polar auxin transport by PIN transporters [18], and (iv) differential growth in elongation regions due to auxin signal-dependent apoplast alkalinization [19]. These results suggest that sensing gravitational stimulation ultimately activates an auxin-regulated signaling network to coordinate directional root growth in response to gravity.

The gene expression in *Arabidopsis* responded to spaceflight, which was altered across a variety of functional protein groups, including the proteins related to heat shock [20], plant defense [21], light response [22], and the cell wall [23]. In *Arabidopsis* roots exposed to micro- or partial gravity, which range from 0.53 g to 0.88 g, the most responsive genes were transcription factors, heat-shock proteins, and defense/cell wall-related proteins [24]. These proteins also fluctuate in plants under salt stress on Earth [25], suggesting that there may be commonalities between intracellular changes during salt stress and in microgravity. In this study, soybeans at an early growth stage were used to clarify the effect of simulated microgravity conditions on salt-stressed plants. Morphological analysis was performed through comparisons between nontreated and salt-stressed soybean in simulated microgravity conditions. Based on the morphological results, a gel- and label-free proteomics was performed to elucidate the mechanisms of the positive effect of simulated microgravity conditions on soybean growth under salt stress. The results of the proteomic analysis were verified for protein accumulation using immunoblot analysis.

## 2. Materials and Methods

### 2.1. Plant Material, Salt Application, and Simulated Microgravity Treatment

**Figure 1:**
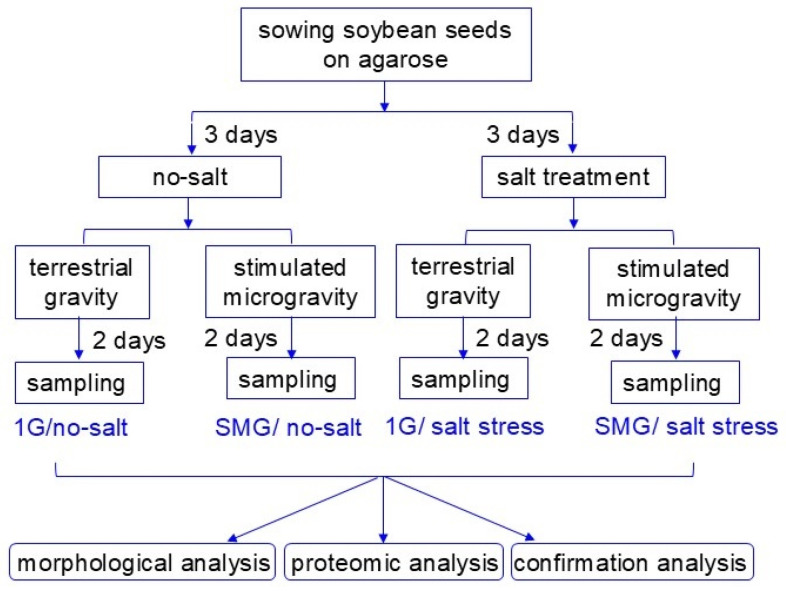
Experimental approach used in this research. Soybean seed was sown onto agarose gel in a tube. Three-day-old soybean plants were treated by applying 2 mL of 150 mM NaCl to the agarose surface. Five tubes with or without 150 mM NaCl application were rotated in a three-dimensional clinostat for 2 days. After treatment with or without simulated microgravity, roots and hypocotyls were collected for each experiment. In the figure, “1G” means terrestrial gravity, and “SMG” means stimulated microgravity. All experiments were carried out in at least three independent biological replicates.

Seeds of soybean (*Glycine max* L. cultivar Enrei) were sterilized with 2% NaClO solution and rinsed twice with water. A seed was sown onto 7 mL of 0.9% agarose gel (Nippon Gene, Tokyo, Japan) in a 15 mL tube and cultivated in a growth chamber, which is at 25 °C, 160 µmol m^−2^ s^−1^, 12 h light/dark conditions. To provoke salt stress based on previous experiments [26], 3-day-old soybean plants were treated by applying 2 mL of 150 mM NaCl (Nacalai Tesque, Kyoto, Japan) to the agarose surface. As control, the same amount of water was applied instead of 150 mL NaCl. Five tubes with or without 150 mM NaCl application were rotated in a three-dimensional clinostat, which simulates a microgravity environment (Figure S1) [27] at 25 °C for 2 days (Figure 1). After treatment with or without simulated microgravity, roots and hypocotyls were collected for each experiment (Figure 1). Proteomic analysis was performed based on the results of morphological analysis, and the proteomic results were verified by immunoblot analysis. In this study, there were four groups: gravity/no salt, stimulated microgravity/no salt, gravity/salt treatment, and stimulated microgravity/salt treatment (Figure 1). At least three independent experiments were performed as biological replications for every experiment with five plants for each replicate, for which the seeds were sown on different days.

### 2.2. Protein Extraction and Concentration Measurement

A portion (500 mg) of samples was ground in 500 µL of extraction buffer consisting of 100 mM NaCl, 50 mM Tris-HCl (pH 7.6), 0.1% SDS, 1% Nonidet-P40, and protease inhibitors (Nacalai Tesque) with a mortar and pestle. The suspension was centrifuged twice with 16,000× *g* for 10 min at 4 °C, and the supernatant was used as the soluble fraction. Protein concentrations were measured for absorbance at 595 nm by the Bradford assay [28] using standardized protein solutions of bovine serum albumin.

### 2.3. Protein Enrichment, Reduction, Alkylation, and Digestion

Quantified protein (100 µg) was adjusted to a final volume of 100 µL, added with 400 µL of methanol, and mixed with 300 µL of water and 100 µL of chloroform. After centrifugation at 16,000× *g* for 10 min, the supernatant was discarded, and 300 µL of methanol was added. After centrifugation with the same conditions, the precipitate was resuspended in 50 mM NH_4_HCO_3_. Proteins were reduced with 50 mM dithiothreitol for 30 min at 56 °C, alkylated with 50 mM iodoacetamide for 30 min at 37 °C in the dark, and digested with lysyl endopeptidase and trypsin (Promega, Madson, WI, USA) at an enzyme/protein ratio of 1:100 for 20 h at 37 °C [29]. Peptide solution was desalted onto a MonoSpin C18 columns (GL Sciences, Tokyo, Japan) and acidified with 1% CF_3_COOH.

### 2.4. Protein Identification Using nanoLC-MS/MS

Nano-liquid chromatography (LC) (EASY-nLC 1000; Thermo Fisher Scientific, Waltham, MA, USA) conditions and mass spectrometry (MS) (Orbitrap Fusion ETD MS; Thermo Fisher Scientific) acquisition settings were described in the previous study [30] (Table S1).

### 2.5. Analysis of MS Data

An MS/MS search was performed with MASCOT (version 2.6.2, Matrix Science, London, UK) and SEQUEST HT search algorithms using the *Arabidopsis thaliana* database (UniProtKB TaxID = 3702) (version 20230503) and *Glycine max* database (UniProtKB TaxID = 3847) (version 20231026), implementing Proteome Discoverer (version 2.4.1.15; Thermo Fisher Scientific). MASCOT settings were detailed in the earlier study [31] (Table S1).

### 2.6. Comparative Analysis of Proteins Using MS Data

Proteome Discoverer with precursor ions quantifiler nodes was used for label-free quantification. Using Perseus (version 1.6.15.0), which a free software, the relative abundance of proteins and peptides was compared between samples [32]. Comparative analysis was detailed in the earlier study [31] (Table S1). Significance was assessed using Student’s *t*-test analysis, and a *p*-value of less than 0.05 was noted as statistically significant. BLAST (version 2.15.0) query against the Gene-Ontology database (http://www.geneontology.org/ (accessed on 10 February 2025); version 22-06-2023) was used for the sequences of differentially accumulated proteins. Function of proteins were classified using MapMan bin codes [33]. Proteins were envisioned using MapMan software (version 3.6.0RC1) [34]. The software and mapping file were downloaded from the MapMan website (http://mapman.gabipd.org/web/guest/mapman (accessed on 10 February 2025); version 10-01-2025).

### 2.7. Analysis Using Immunoblotting

Quantified protein (10 µg) was supplemented with sample buffer containing 2% SDS, 62.5 mM Tris-HCl (pH 6.8), 5% dithiothreitol, bromophenol blue, and 10% glycerol (Bio-Rad, Hercules, CA, USA) [35] and separated by electrophoresis on a 10% SDS-polyacrylamide gel. Proteins in gel were stained with Coomassie brilliant blue as a loading control. On the other hand, proteins in gel were transferred to a polyvinylidene difluoride (PVDF) membrane using a semidry transfer blotter. The PVDF membrane was blocked with Bullet Blocking One regent (Nacalai Tesque) for 10 min and cross-reacted with anti-osmotin [36], Cu/Zn superoxide dismutase (SOD) (Proteintech, Rosemont, IL, USA), ascorbate peroxidase (APX) [37], aquaporin (Cosmo Bio, Tokyo, Japan), and calnexin [38] antibodies as the primary antibodies for 30 min. As the secondary antibody, anti-rabbit IgG conjugated with horseradish peroxidase (Bio-Rad) was used. After a cross-reaction of 30 min, signals were detected using 3,3′,5,5′-tetramethylbenzidine membrane peroxidase substrate system (SeraCare, Gaithersburg, MD, USA). The integrated density of the bands was calculated with ImageJ software (version 1.53e with Java 1.8.0_172; National Institutes of Health, Bethesda, MD, USA).

### 2.8. Statistical Analysis

Data from morphological and immunoblot analyses were assessed by one-way ANOVA and subsequently Tukey’s multiple comparison among groups using SPSS (version 29; IBM, Chicago, IL, USA). A *p*-value of less than 0.05 was noted as statistically significant.

## 3. Results

### 3.1. Morphological Changes of Soybean Induced by Simulated Microgravity Condition Under Salt Stress

**Figure 2:**
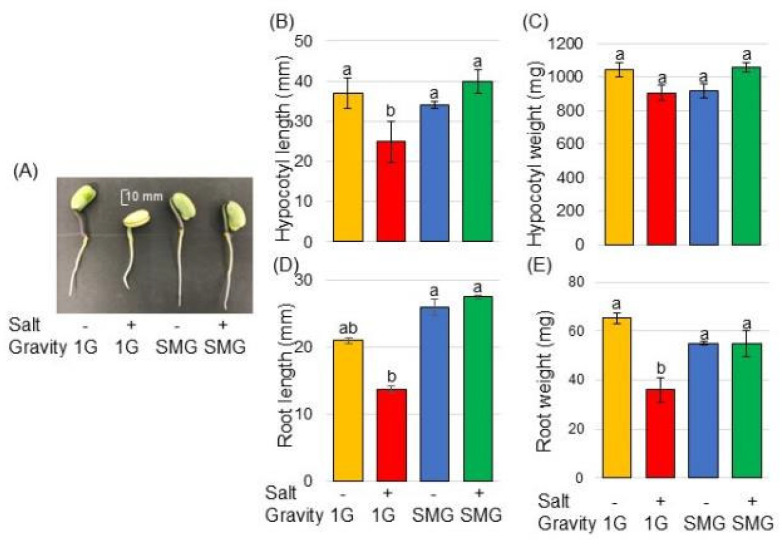
Morphological effect of simulated microgravity condition on soybean under salt stress. Soybean seed was sown onto agarose gel in a tube. Three-day-old soybean plants were treated with or without salt stress on the agarose surface. Tubes were treated with or without simulated microgravity for 2 days. Size bar in the picture shows 10 mm (**A**). As morphological parameters, hypocotyl length (**B**), hypocotyl fresh weight (**C**), main-root length (**D**), and total root fresh weight (**E**) were measured. The data are provided as the mean ± SD from six independent biological replicates. The mean values of each point with different letters are significant according to one-way ANOVA followed by Tukey’s multiple comparisons (*p* < 0.05).

Morphological changes were analyzed to investigate the effects of simulated microgravity on soybeans under salt stress. Three-day-old soybean plants on agarose gel were treated with or without salt stress and with or without rotation of a 3-dimensional clinostat for 2 days (Figure 1). The length and fresh weight of hypocotyl as well as main-root length and total root fresh weight were measured as morphological parameters (Figure 2). Roots tended to grow longer under simulated microgravity conditions than when gravity was present (Figure 2D). Under gravity, salt stress significantly reduced the root weight and hypocotyl length (Figure 2B,E), whereas salt stress did not suppress those parameters under simulated microgravity conditions (Figure 2B,D,E). Because morphological measurements showed that the effects of salt stress and microgravity were more pronounced in roots than in hypocotyls, the root of soybean was selected for proteomic analysis.

### 3.2. Identification and Functional Classification of Proteins in Soybean Root Treated with Simulated Microgravity Under Salt Stress

**Figure 3:**
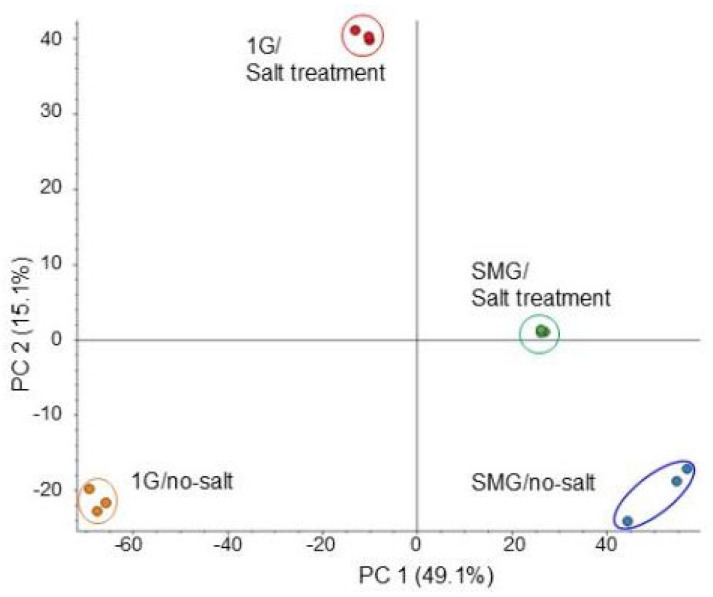
Summary of whole proteomic data from 12 samples of soybean root based on principal-component analysis. Three-day-old soybean plants were treated with or without simulated microgravity condition with or without salt stress for 2 days. Roots were used for LC-MS/MS analysis. Proteomics was performed on three independent biological replicates for each treatment. Principal component analysis was conducted with Proteome Discoverer.

To investigate the cellular mechanisms on soybean growth in simulated microgravity condition under salt stress, a gel- and label-free proteomics was performed on the root. Three-day-old soybean plants on agarose gel were treated with or without salt stress and with or without rotation of a 3-dimensional clinostat for 2 days (Figure 1). The proteins extracted from root were enriched, reduced, alkylated, digested, and analyzed using nanoLC-MS/MS. After analysis, the relative abundance of proteins was compared with each other, and a total of 5236 and 1271 proteins were detected using the protein databases of *Glycine max* and *Arabidopsis thaliana*, respectively. Many proteins were identified by using the *Glycine max* database in comparison with the *Arabidopsis thaliana* database, so the results of the *Glycine max* database were used in this study. Comparison of the proteomic results from all 12 samples in four groups by principal component analysis revealed distinct protein-accumulation patterns from the four different treatments (Figure 3). The results showed that the identified proteins were distributed in different groups among the three treatment groups compared with control, which was gravity/no salt. Additionally, salt stress largely affected soybean root proteins under gravity (Figure 3).

The relative protein abundance in stimulated microgravity was compared with that under gravity (Table 1 and Table S2). The abundance of 1547 proteins was differentially altered in the roots in stimulated microgravity compared with that under gravity (Table S2). Among the 1547 proteins, 276 proteins were increased and 1271 proteins decreased in stimulated microgravity compared to under gravity (Table S2). Among 1547 proteins, proteins related to translation, ribosomal biogenesis, protein folding, oxidative stress, cell organization, and cell wall organization were significantly changed in stimulated microgravity compared with gravity (Table S2). Furthermore, proteins related to phytohormone signaling and ubiquitin/proteasome system were altered in stimulated microgravity compared with gravity (Table 1).

The relative protein abundance of salt-stressed soybeans was compared with the no-salt condition under gravity (Table S3) or under the simulated microgravity condition (Table S4).

**Figure 4:**
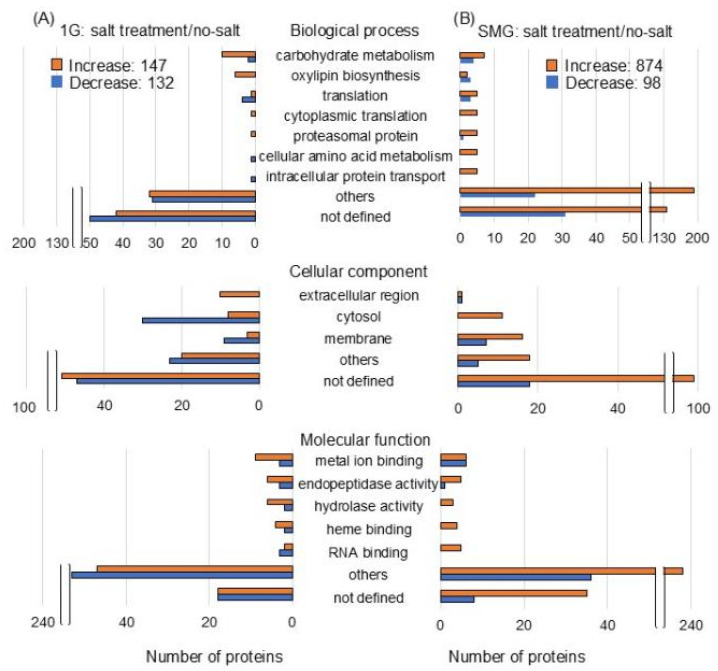
Functional categories of proteins with differential abundance in soybean root exposed to simulated microgravity condition under salt stress. Three-day-old soybean plants were treated with or without simulated microgravity condition with or without salt stress for 2 days. Proteomics was conducted with three independent biological replicates for each treatment. After proteomic analysis, functional categories of significantly changed proteins (*p* < 0.05 by the Student’s *t*-test) were determined using Gene Ontology analysis (Tables S3 and S4). (**A**) salt treatment/no-salt under gravity and (**B**) salt treatment/no-salt under simulated microgravity. Orange and blue columns indicate increased and decreased proteins.

The abundance of 279 and 972 proteins differentially changed in the roots of salt-stressed soybeans compared with the no-salt condition under gravity and the simulated microgravity condition, respectively (Tables S3 and S4). Of the 279 proteins, 147 proteins were increased and 132 proteins decreased with salt stress compared to the no-salt condition under gravity (Figure 4A). Of the 972 proteins, 874 proteins were increased and 98 proteins decreased with salt stress compared to the no-salt condition in the simulated microgravity condition (Figure 4B).

**Figure 5:**
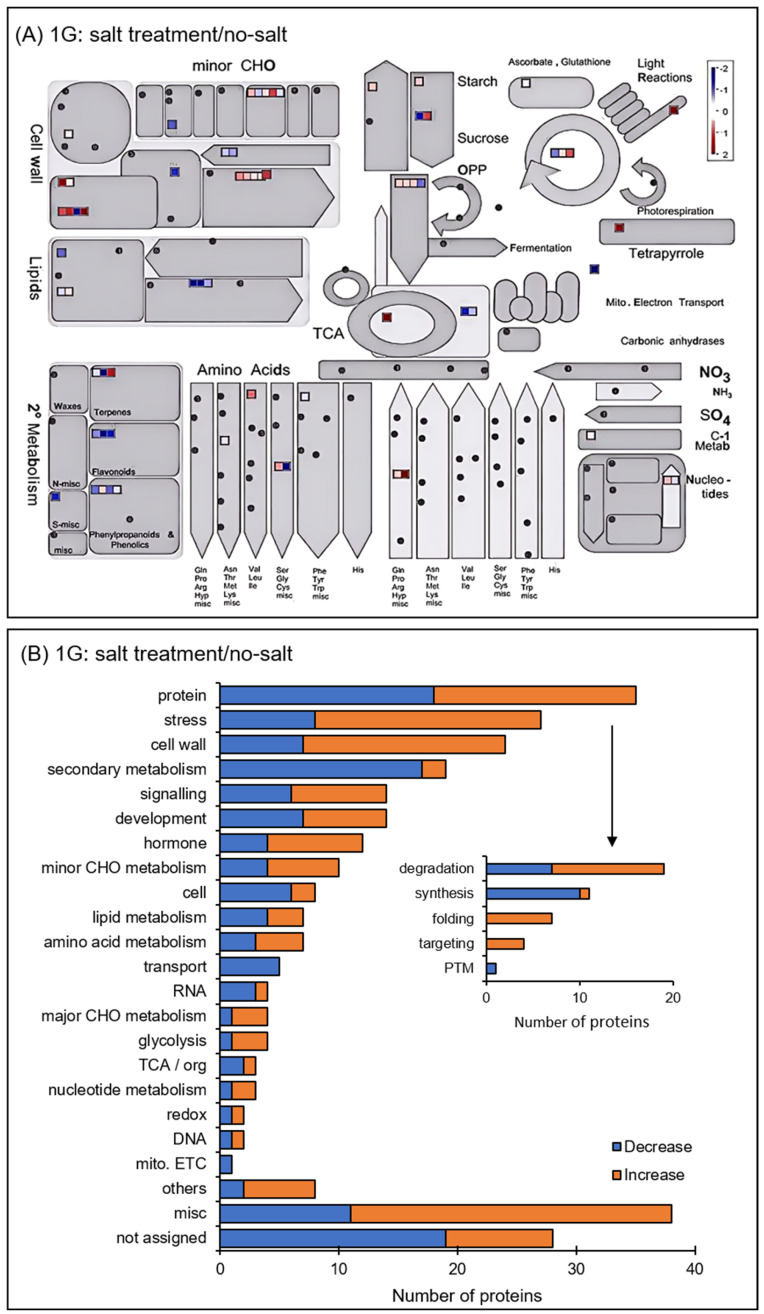
Functional categories of changed proteins under salt stress compared with no-salt condition under gravity. Orange and blue columns indicate increased and decreased proteins with salt stress compared with the no-salt condition under gravity. The abbreviations of the functional category of proteins were determined using MapMan bin codes (**A**) and MapMan software (**B**). The functional categories are as follows: CHO, carbohydrates; cell, cell organization/vesicle transport; RNA, RNA processing/regulation of transcription; TCA, tricarboxylic acid cycle; redox, redox ascorbate/glutathione metabolism; DNA, DNA synthesis/repair; mito. ETC, mitochondrial electron transport; PTM, post-translational modifications. “others” shows protein with other functions; “misc” shows miscellaneous; “not assigned” shows proteins without ontology.

**Figure 6:**
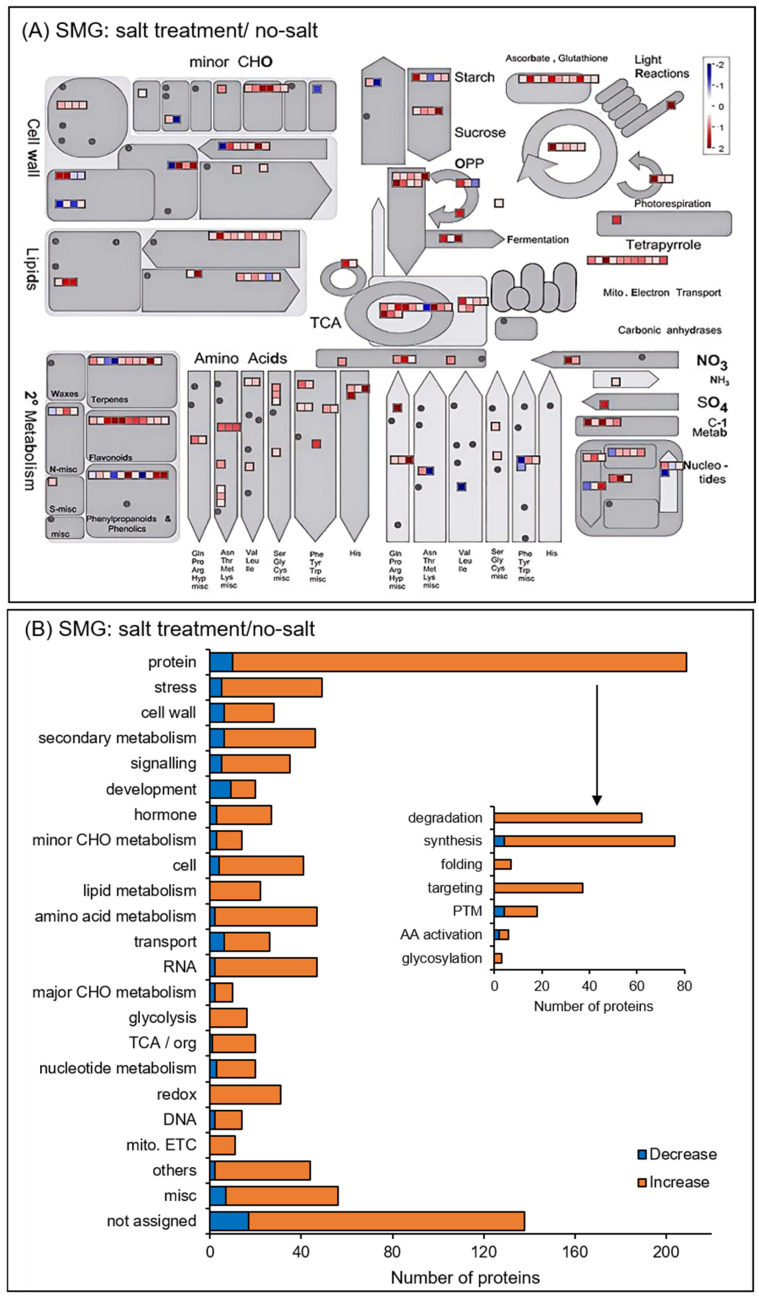
Functional categories of changed proteins under salt stress compared with no-salt condition under simulated microgravity. Orange and blue columns indicate increased and decreased proteins with salt stress compared with no-salt condition under simulated microgravity. The functional category of proteins was determined using MapMan bin codes (**A**) and MapMan software (**B**). The functional category abbreviations are the same as in Figure 5.

The functional categories of identified proteins were obtained using Gene Ontology analysis (Figure 4). Functional classifications of proteins showed opposite trends of increase and decrease due to salt stress under gravity and simulated microgravity condition. They were oxylipin biosynthesis and translation in biological process; extracellular, cytoplasm, and membrane in cellular component; and RNA binding in molecular function (Figure 4). Additionally, the functional categories of proteins were determined using MapMan bin codes and MapMan software (Figure 5 and Figure 6). Functional classifications of proteins, which showed opposite trends of increase and decrease due to salt stress under gravity and the simulated microgravity condition, were protein metabolism, secondary metabolism, and redox-related proteins (Figure 5 and Figure 6).

### 3.3. Protein Accumulation Altered in Soybean Treated with Simulated Microgravity Under Salt Stress

**Figure 7:**
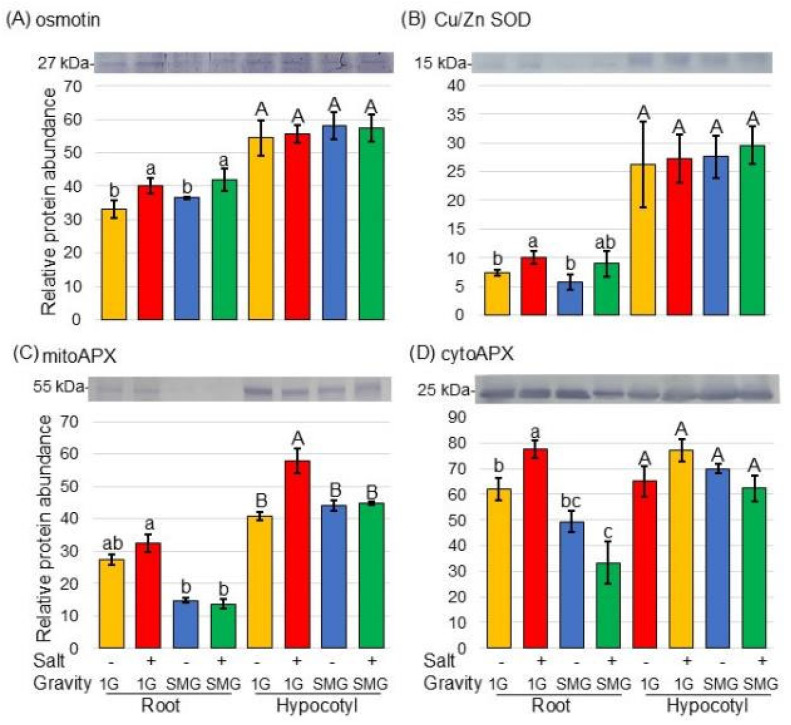
Immunoblot analysis of proteins changed in the ROS-scavenging system of soybean in microgravity conditions under salt stress. Soybean seeds were sown on the agarose, and seedlings were treated with or without salt stress as well as in microgravity and gravity. Proteins extracted from roots and hypocotyls were separated by SDS-polyacrylamide gel electrophoresis. Coomassie brilliant blue staining pattern was used as a loading control (Figure S2). Proteins transferred onto PVDF membranes were cross-reacted with anti-osmotin (**A**), Cu/Zn SOD (**B**), and APX (**C**,**D**) antibodies. (**A**) osmotin, (**B**) Cu/Zu SOD, (**C**) mitoAPX, and (**D**) cytoAPX. The integrated density of the bands was calculated with ImageJ software. Data are shown as the mean ± SD from three independent biological replicates (Figures S3–S5). Statistical analysis is the same as in Figure 2. Lowercase letters indicate the significance when comparing roots, and uppercase letters indicate the significance when comparing hypocotyls.

First, to confirm whether soybeans were exposed to salt stress, the accumulation of osmotin, which is a salt stress-responsive protein, was examined using immunoblot analysis. Proteins extracted from root and hypocotyl of soybean, which was treated with or without salt stress and with or without rotation in a 3-dimensional clinostat, were separated on SDS-polyacrylamide gel electrophoresis. The Coomassie brilliant blue staining pattern was used as a loading control (Figure S2). The abundance of osmotin, which is a marker protein for response to salt stress, increased in soybean roots under salt stress and was restored by simulated microgravity conditions. (Figure 7A and Figure S3).

Because proteomic analysis revealed changes in the amounts of reactive oxygen species (ROS)-scavenging proteins (Figure 5 and Figure 6), the changes in protein amounts were confirmed by immunoblot analysis (Figure 7B–D). SOD and cytoAPX increased in soybean root under salt stress; however, they did not increase in simulated microgravity conditions even under salt stress (Figure 7B,D, Figures S4 and S5). Furthermore, mitoAPX increased in soybean root and hypocotyl under salt stress; however, it did not increase in simulated microgravity conditions even under salt stress (Figure 7C and Figure S5).

**Figure 8:**
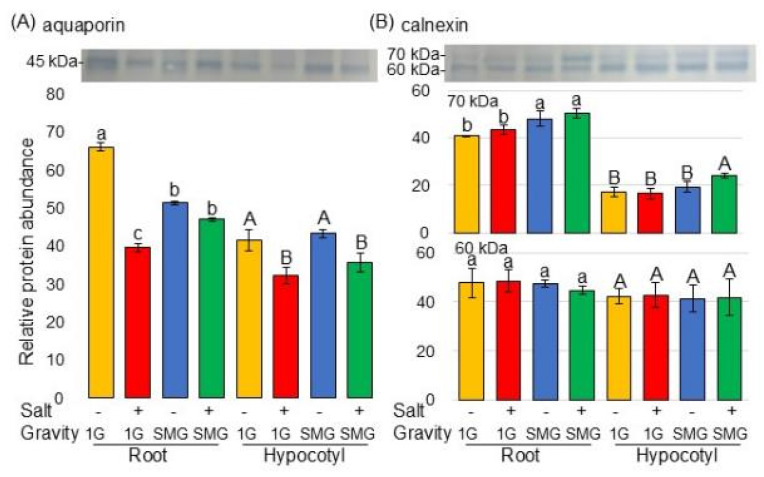
Immunoblot analysis of proteins changed in the membrane of soybean in microgravity condition under salt stress. Sample processing and electrophoresis methods were the same as in Figure 7. Coomassie brilliant blue staining pattern was used as a loading control (Figure S2). Proteins transferred onto PVDF membranes were cross-reacted with anti-aquaporin (**A**) and calnexin (**B**) antibodies. The integrated density of the bands was calculated with ImageJ software. Data are shown as the mean ± SD from three independent biological replicates (Figures S6 and S7). Statistical analysis is the same as in Figure 2. Lowercase letters indicate the significance when comparing roots, and uppercase letters indicate the significance when comparing hypocotyls.

Examination of previous papers revealed that calnexin and aquaporin fluctuate under microgravity. In this study, the proteomic analysis showed significant changes in membrane proteins (Figure 4), but calnexin and aquaporin were not detected in proteomic data (Tables S3 and S4). Therefore, to investigate whether these proteins were truly unchanged in soybeans, the changes in protein amounts were confirmed by immunoblot analysis (Figure 8). The amount of 45 kDa aquaporin significantly decreased in the root and hypocotyl of soybean under salt stress; however, in the root, it recovered in simulated microgravity conditions even under stress (Figure 8A and Figure S6). The amount of 70 kDa calnexin in soybean root increased in simulated microgravity conditions with or without salt stress (Figure 8B and Figure S7). On the other hand, 60 kDa calnexin did not change in any conditions (Figure 8B and Figure S7).

## 4. Discussion

### 4.1. Simulated Microgravity Mitigates the Negative Growth Effects of Salt in Soybean

Exposure to micro- or partial gravity, which is a stressor and affects plant growth, alters physiological functions and activates the stress response of plants. Plant roots grow underground in the direction of gravity, and plants have evolved under the constant level of gravitational acceleration on Earth [39,40,41]. The growth of *Arabidopsis* seedling and rice root was improved under microgravity [42,43]. In the RICE (BRIC-RC) experiment on the Space Shuttle STS-95, microgravity promoted the elongation of *Arabidopsis* hypocotyls and rice coleoptiles [44,45]. In the Aniso-Tubule (CBEF) experiment on the Space Shuttle Kibo, a microgravity environment enhanced the elongation of *Arabidopsis* hypocotyls, while it suppressed thickening [46]. These previous studies indicate that the growth of plants such as *Arabidopsis* and rice is promoted in a microgravity environment. In the soybeans used in this study, roots also tended to grow longer in the simulated microgravity environment than when gravity was present (Figure 2). Additionally, salt stress significantly reduced the length of roots and hypocotyls under gravity, whereas under simulated microgravity conditions, salt stress did not suppress their length (Figure 2). The present result and previous findings suggest that the elongation of soybean root in agarose gel is enhanced in simulated microgravity conditions compared with gravity conditions, similar to rice and *Arabidopsis*. Furthermore, this result proposes that soybean roots in simulated microgravity conditions might be insensitive to Na ions.

### 4.2. Simulated Microgravity Treatment Has Positive Effect on Soybean Growth Through Phytohormone

The levels and profiles of key phytohormones did not significantly change under the microgravity environment in space, but gene-expression analysis revealed changes in auxin and ethylene metabolism in rice [47]. In this study, proteins related to the metabolism of auxin, abscisic acid, and jasmonic acid were altered in simulated microgravity conditions compared with gravity (Table 1). It was reported that outer space conditions significantly increased proteins associated with auxin metabolism and transport [48]. Seedlings treated with auxin-transport inhibitor produced 50% less under clinorotation ethylene than untreated control receiving identical gravity treatment, whereas treatment with 2,4-dichlorophenoxyacetic acid produced 5-fold ethylene when plants were clinorotated [49]. Relative to onboard gravity, genes taking part in transcriptional regulation, shoot development, and auxin response along with light were upregulated by microgravity [50]. The endogenous levels of indole-3-acetic acid in seedlings grown under microgravity in space were substantially higher than those on Earth [51]. These reports imply that auxin is involved in plant growth in a microgravity environment. The present result with these findings suggests that phytohormone biosynthesis and transport may be involved in soybean root growth under microgravity.

### 4.3. Simulated Microgravity Treatment Decreases ROS Production Even Under Salt Stress

Controlling the accumulation of ROS, antioxidants, and oxidoreductases in plant cells is important for plant survival. ROS are generated in systemic signaling and direct stress metabolism in plants. The observation of conserved roles for COR78 and HSP101 in *Arabidopsis* indicated that severe disruption of plastid-associated ROS and antioxidant systems accompanied plant growth in space [52]. *Arabidopsis* cells accumulated ROS- and calcium-related proteins [53], and comparable results were reported in simulated microgravity experiments [54]. Experiments using mainly *Arabidopsis* plant tissue cultures or seedlings as experimental materials revealed the potential contributions of light, ROS, and calcium signaling in plant acclimatization to the spaceflight environment.

During spaceflight, a large number of genes and proteins involved in maintaining ROS signaling and cellular redox homeostasis were significantly altered [55]. The accumulation of ROS and polyphenols counteracted oxidative damage and maintained the integrity of important structures in *Brassica rapa* microgreens [56]. In this study, SOD and APXs as ROS-scavenging proteins increased in soybean root under salt stress; however, they did not increase in simulated microgravity conditions even under stress (Figure 5 and Figure 6). Many studies using *Arabidopsis* in space have reported that ROS increased; however, ROS-scavenging systems were not altered in soybean using a clinostat. The reason might be that soybeans were sown on agarose, which reduced environmental stimuli in this study.

### 4.4. Simulated Microgravity Treatment Alters Membrane-Localized Proteins

The unexpected reduction in aquaporins observed in plants grown in space conditions could be related to differences in water in plants present on the International Space Station and in the European Modular Cultivation System on Earth [57]. Although there were no statistically significant differences between microgravity and artificial gravity environments, the accumulation of NIP3-1/ plasma membrane intrinsic protein11 and aquaporin1/tonoplast intrinsic protein tended to increase and decrease, respectively [58]. In this study, the accumulation of aquaporin in soybean root under salt stress increased in simulated microgravity conditions compared to under gravity (Figure 5). This and previous findings suggest a possible role for aquaporin in controlling the growth of plants growing under the microgravity conditions of space.

Calnexin is a transmembrane protein that functions as a chaperone in the endoplasmic reticulum to ensure the correct folding of proteins under stress conditions, allowing organisms to withstand harmful conditions [59]. Endoplasmic reticulum stress due to activation of misfolded protein responses was identified by increased levels of calnexin, protein disulfide isomerase, BiP/GRP78, and ERO1-Lα [60]. In previous studies, 60 kDa calnexin was not altered by any treatment, whereas 70 kDa calnexin increased by flooding stress compared to the control. Furthermore, after this accumulation, millimeter wave-irradiated wheat recovered to the control level even under flooding stress [61]. In this study, although 60 kDa calnexin was not altered by any treatment, the accumulation of 70 kDa calreticulin in soybean root under salt stress increased in simulated microgravity conditions compared to under gravity conditions (Figure 5 and Figure 7). Phosphorylation of the cytoplasmic tail of calnexin regulates calcium ATPase in the endoplasmic reticulum, and calcium moves into the endoplasmic reticulum. Its expression under various stress conditions provides insights into the crosstalk between endoplasmic reticulum stress and abiotic stress signaling [62]. Calnexin is a phosphorylated protein, which is present in large quantities in the membranes of the endoplasmic reticulum of eukaryotic cells [63]. Based on these reports, the 70 kDa calnexin may be a molecular weight alteration resulting from phosphorylation of the 60 kDa calnexin. These reports, taken together with the present results, suggest that calnexin might be phosphorylated to regulate the misfolding of proteins in soybean root in simulated microgravity through the reduction in misfolded proteins.

## 5. Conclusions

To determine the effect of simulated microgravity conditions on salt-stressed plants, soybeans were used at an early growth stage. Roots grew more under simulated microgravity conditions than in the presence of gravity; additionally, root shortening due to salt stress was not inhibited under simulated microgravity conditions. To elucidate the salt stress-tolerant mechanism in soybean root under simulated microgravity environment, a proteomic analysis was conducted. The results of the verification experiment revealed the following: (i) SOD and APXs increased in soybean root under salt stress; however, they did not increase in simulated microgravity conditions even under salt stress, and (ii) 45 kDa aquaporin and 70 kDa calnexin in soybean root under salt stress increased in simulated microgravity conditions compared to under gravity conditions. These results suggest that soybean growth under salt stress might be modulated in simulated microgravity conditions through improving water permeability, mitigating ROS production, and restoring protein folding.

## Figures and Tables

**Table 1:** The list of significantly altered proteins under stimulated microgravity compared with gravity, which were selected from Table S2.

Difference	Accession	Protein Names	Gene Ontology
Phytohormone-related proteins			
0.6143	F6KBT6	Allene oxide cyclase 6	Jasmonic acid biosynthetic process
−0.8150	I1LY51	Oxidored_FMN protein	Jasmonic acid biosynthetic process
−2.6814	I1JNN8	Dirigent protein	Jasmonic acid biosynthetic process
−0.9307	A0A0R0H3J2	Serine/threonine-protein phosphatase	Brassinosteroid mediated signaling pathway
−1.5529	I1JJV5	Protein kinase	Brassinosteroid mediated signaling pathway
0.5960	C6TIU6	7-dehydrocholesterol reductase	Brassinosteroid biosynthetic process
−2.0043	K7MV36	UBR-type domain-containing protein	Auxin polar transport
−0.6056	D3G9M7	Calcium dependent protein kinase	Abscisic acid-activated signaling pathway
−1.2503	I1K6C6	Serine/threonine-protein kinase	Abscisic acid-activated signaling pathway
−3.8083	I1KBT7	Calcium-dependent protein kinase	Abscisic acid-activated signaling pathway
−1.5482	E6YBW4	Pathogenesis-related protein 10	Abscisic acid-activated signaling pathway
−1.2505	I1MRM5	Bet_v_1 domain-containing protein	Abscisic acid-activated signaling pathway
−0.6948	I1KE09	Bet_v_1 domain-containing protein	Abscisic acid-activated signaling pathway
Ubiquitin/proteasome-related proteins			
0.6168	C6TG97	Proteasome subunit alpha type	Ubiquitin-dependent protein
−0.8034	I1NB01	MPN domain-containing protein	Ubiquitin-dependent protein
−1.0399	I1K657	RPN13_C domain-containing protein	Ubiquitin-dependent protein
−1.3676	I1J8D4	Ubiquitin-dependent protein	Ubiquitin-dependent protein
−1.4256	I1K1Z8	PCI domain-containing protein	Ubiquitin-dependent protein
−1.5579	I1JCA6	Uncharacterized protein	Ubiquitin-dependent protein
−1.6108	C6T2F1	UBIQUITIN_CONJUGAT_2	Ubiquitin-dependent protein
−3.1095	I1JQF4	MPN domain-containing protein	Ubiquitin-dependent protein
0.9386	C6T574	Ubiquitin carboxyl-terminal hydrolase	Protein deubiquitination
−0.6865	A0A0R0L2F3	Ubiquitinyl hydrolase 1	Protein deubiquitination
−0.8156	I1KGF2	Ubiquitin carboxyl-terminal hydrolase	Protein deubiquitination
−1.1460	I1NIJ5	Ubiquitin carboxyl-terminal hydrolase	Protein deubiquitination
−1.7558	I1LSZ2	Ubiquitin carboxyl-terminal hydrolase	Protein deubiquitination
−1.4141	I1L9D9	USP domain-containing protein	Protein deubiquitination
−1.5029	I1MF26	CULLIN_2 domain-containing protein	Proteasome-ubiquitin-dependent protein
−1.3441	K7KAA3	RING-type domain-containing protein	Proteasome-ubiquitin-dependent protein
−0.6033	I1LS70	26S proteasome non-ATPas	Proteasome-ubiquitin-dependent protein
−0.8114	I1KQE8	26S proteasome non-ATPase	Proteasome-ubiquitin-dependent protein
−1.7403	I1K5H2	26S proteasome non-ATPase	Proteasome-ubiquitin-dependent protein
−0.7464	I1LMP2	MPN domain-containing protein	Proteasome-ubiquitin-dependent protein
−0.7495	I1J8K2	VWFA domain-containing protein	Proteasome assembly
−0.8726	I1JCF9	VWFA domain-containing protein	Proteasome assembly
−0.6417	I1KDR0	26S proteasome non-ATPase	Proteasome assembly
−2.4357	A0A0R0G8R3	Proteasome component Ecm29	Proteasome assembly
−1.3209	I1N058	Proteasome activator subunit 4	Proteasomal ubiquitin catabolic process
0.8760	I1JBH2	Proteasome subunit alpha type	Proteasomal protein catabolic process
0.6471	I1N783	Proteasome subunit alpha type	Proteasomal protein catabolic process
−0.6180	I1MA63	Proteasome subunit beta	Proteasomal protein catabolic process
−0.7445	A0A0R4J2M8	Proteasome subunit alpha type	Proteasomal protein catabolic process

Orange and blue colors indicate increased and decreased proteins, respectively.

## Data Availability

For MS data, the RAW data, peak lists, and result files have been deposited in the ProteomeXchange Consortium [64] via the jPOST [65] partner repository under data-set identifiers PXD048989.

## Supplementary Materials

The following supporting information can be downloaded at https://www.mdpi.com/article/10.3390/cells14070541/s1. Table S1: Protein identification using nanoLC-MS/MS, MS data analysis, and differential analysis of proteins using MS data; Table S2: List of significantly changed proteins in soybean roots in simulated microgravity condition compared with gravity; Table S3: List of significantly changed proteins in soybean roots in gravity under salt stress compared with no-salt condition; Table S4: List of significantly changed proteins in soybean roots in simulated microgravity condition under salt stress compared with no-salt condition; Figure S1: Device for simulated microgravity condition.; Figure S2: The Coomassie brilliant blue staining pattern of proteins used for immunoblot analysis; Figure S3: Blots of the entire PVDF membrane with anti-osmotin antibody, which is used in Figure 7A; Figure S4: Blots of the entire PVDF membrane with anti-SOD antibody, which is used in Figure 7B; Figure S5: Blots of the entire PVDF membrane with anti-APX antibody, which is used in Figure 7C,D; Figure S6: Blots of the entire PVDF membrane with anti-aquaporin antibody, which is used in Figure 8A; Figure S7: Blots of the entire PVDF membrane with anti-calnexin antibody, which is used in Figure 8B.

## Abbreviations

The following abbreviations are used in this manuscript:

LC	Liquid chromatography
MS	Mass spectrometry
ROS	Reactive oxygen species
SOD	Superoxide dismutase
APX	Ascorbate peroxidase

## References

[1] Muhammad A., Khan M.H.U., Kong X., Zheng S., Bai N., Li L., Zhang N., Muhammad S., Li Z., Zhang X. (2025). Rhizospheric crosstalk: A mechanistic overview of how plant secondary metabolites alleviate abiotic stresses. Plant Sci..

[2] Singh A. (2021). Soil salinization management for sustainable development: A review. J. Environ. Manag..

[3] Golldack D., Li C., Mohan H., Probst N. (2014). Tolerance to drought and salt stress in plants: Unraveling the signaling networks. Front. Plant Sci..

[4] Zhou H., Shi H., Yang Y., Feng X., Chen X., Xiao F., Lin H., Guo Y. (2024). Insights into plant salt stress signaling and tolerance. J. Genet. Genom..

[5] Yuan F., Leng B., Wang B. (2016). Progress in studying salt secretion from the salt glands in recretohalophytes: How do plants secrete salt?. Front. Plant Sci..

[6] Zhu J.-K. (2003). Regulation of ion homeostasis under salt stress. Curr. Opin. Plant Biol..

[7] Arif Y., Singh P., Siddiqui H., Bajguz A., Hayat S. (2020). Salinity induced physiological and biochemical changes in plants: An omic approach towards salt stress tolerance. Plant Physiol. Biochem..

[8] Kronzucker H.J., Britto D.T. (2011). Sodium transport in plants: A critical review. New Phytol..

[9] Kronzucker H.J., Coskun D., Schulze L.M., Wong J.R., Britto D.T. (2013). Sodium as nutrient and toxicant. Plant Soil.

[10] Mo M., Yokawa K., Wan Y., Baluška F. (2015). How and why do root apices sense light under the soil surface?. Front. Plant Sci..

[11] Lynch J.P., Brown K.M. (2012). New roots for agriculture: Exploiting the root phenome. Philos. Trans. R. Soc. B Biol. Sci..

[12] de Dorlodot S., Forster B., Pagès L., Price A., Tuberosa R., Draye X. (2007). Root system architecture: Opportunities and constraints for genetic improvement of crops. Trends Plant Sci..

[13] Zhang H., Liu Z., Zheng C., Ma H., Zeng M., Yang X. (2025). Root system architecture plasticity with beneficial rhizosphere microbes: Current findings and future perspectives. Microbiol. Res..

[14] Jan M., Muhammad S., Jin W., Zhong W., Zhang S., Lin Y., Zhou Y., Liu J., Liu H., Munir R. (2024). Modulating root system architecture: Crosstalk between auxin and phytohormones. Front. Plant Sci..

[15] Kuya N., Nishijima R., Kitomi Y., Kawakatsu T., Uga Y. (2023). Transcriptome profiles of rice roots under simulated microgravity conditions and following gravistimulation. Front. Plant Sci..

[16] Nakamura M., Nishimura T., Morita M.T. (2019). Gravity sensing and signal conversion in plant gravitropism. J. Exp. Bot..

[17] Furutani M., Hirano Y., Nishimura T., Nakamura M., Taniguchi M., Suzuki K., Oshida R., Kondo C., Sun S., Kato K. (2020). Polar recruitment of RLD by LAZY1-like protein during gravity signaling in root branch angle control. Nat. Commun..

[18] Han H., Adamowski M., Qi L., Alotaibi S.S., Friml J. (2021). PIN-mediated polar auxin transport regulations in plant tropic responses. New Phytol..

[19] Li L., Chen H., Alotaibi S.S., Pěnčík A., Adamowski M., Novák O., Friml J. (2022). RALF1 peptide triggers biphasic root growth inhibition upstream of auxin biosynthesis. Proc. Natl. Acad. Sci. USA.

[20] Choi W.G., Barker R.J., Kim S.H., Swanson S.J., Gilroy S. (2019). Variation in the transcriptome of different ecotypes of *Arabidopsis thaliana* reveals signatures of oxidative stress in plant responses to spaceflight. Am. J. Bot..

[21] Fengler S., Spirer I., Neef M., Ecke M., Nieselt K., Hampp R. (2015). A whole-genome microarray study of *Arabidopsis thaliana* semisolid callus cultures exposed to microgravity and nonmicrogravity related spaceflight conditions for 5 days on board of Shenzhou 8. BioMed Res. Int..

[22] Vandenbrink J.P., Herranz R., Poehlman W.L., Alex Feltus F., Villacampa A., Ciska M., Javier Medina F., Kiss J.Z. (2019). RNA-seq analyses of *Arabidopsis thaliana* seedlings after exposure to blue-light phototropic stimuli in microgravity. Am. J. Bot..

[23] Johnson C.M., Subramanian A., Pattathil S., Correll M.J., Kiss J.Z. (2017). Comparative transcriptomics indicate changes in cell wall organization and stress response in seedlings during spaceflight. Am. J. Bot..

[24] Sheppard J., Land E.S., Toennisson T.A., Doherty C.J., Perera I.Y. (2021). Uncovering transcriptional responses to fractional gravity in *Arabidopsis* roots. Life.

[25] Wang X., Wang Y., Jiang Y., Wang H., Zhou L., Li F., Wang L., Jiang D., Chen F., Chen S. (2024). Transcription factor CmHSFA4-CmMYBS3 complex enhances salt tolerance in chrysanthemum by repressing CmMYB121 expression. Plant Physiol..

[26] Komatsu S., Nishiuchi T. (2024). Proteomic Analysis to Understand the Promotive Effect of Ethanol on Soybean Growth under Salt Stress. Biology.

[27] Takahashi A., Yamanouchi S., Takeuchi K., Takahashi S., Tashiro M., Hidema J., Higashitani A., Adachi T., Zhang S., Guirguis F.N.L. (2020). Combined environment simulator for low-dose-rate radiation and partial gravity of moon and mars. Life.

[28] Bradford M.M. (1976). A rapid and sensitive method for the quantitation of microgram quantities of protein utilizing the principle of protein-dye binding. Anal. Biochem..

[29] Komatsu S., Han C., Nanjo Y., Altaf-Un-Nahar M., Wang K., He D., Yang P. (2013). Label-free quantitative proteomic analysis of abscisic acid effect in early-stage soybean under flooding. J. Proteome Res..

[30] Li X., Rehman S.U., Yamaguchi H., Hitachi K., Tsuchida K., Yamaguchi T., Sunohara Y., Matsumoto H., Komatsu S. (2018). Proteomic analysis of the effect of plant-derived smoke on soybean during recovery from flooding stress. J. Proteom..

[31] Komatsu S., Maruyama J., Furuya T., Yin X., Yamaguchi H., Hitachi K., Miyashita N., Tsuchida K., Tani M. (2021). Proteomic and biological analyses reveal the effect on growth under flooding stress of chickpea irradiated with millimeter waves. J. Proteome Res..

[32] Tyanova S., Temu T., Sinitcyn P., Carlson A., Hein M.Y., Geiger T., Mann M., Cox J. (2016). The Perseus computational platform for comprehensive analysis of omics data. Nat. Methods.

[33] Usadel B., Nagel A., Thimm O., Redestig H., Blaesing O.E., Palacios Rofas N., Selbig J., Hannemann J., Piques M.C., Steinhauser D. (2005). Extension of the visualization tool mapman to allow statistical analysis of arrays, display of corresponding genes, and comparison with known databases. Plant Physiol..

[34] Usadel B., Poree F., Nagel A., Lohse M., Czedik-Eysenberg A., Stitt M. (2009). A guide to using mapman to visualize and compare omics data in plants: A case study in the crop species, maize. Plant Cell Environ..

[35] Laemmli U.K. (1970). Cleavage of structural proteins during the assembly of the head of bacteriophage T4. Nature.

[36] Konishi H., Ishiguro K., Komatsu S. (2001). A proteomics approach towards understanding blast fungus infection of rice grown under different levels of nitrogen fertilization. Proteomics.

[37] Komatsu S., Yamamoto A., Nakamura T., Nouri M.Z., Nanjo Y., Nishizawa K., Furukawa K. (2011). Comprehensive analysis of mitochondria in roots and hypocotyls of soybean under flooding stress using proteomics and metabolomics techniques. J. Proteome Res..

[38] Komatsu S., Masuda T., Abe K. (1996). Phosphorylation of a protein (pp56) is related to the regeneration of rice cultured suspension cells. Plant Cell Physiol..

[39] Villacampa A., Sora L., Herranz R., Medina F.J., Ciska M. (2021). Analysis of graviresponse and biological effects of vertical and horizontal clinorotation in *Arabidopsis thaliana* root tip. Plants.

[40] Millar K.D.L., Johnson C.M., Edelmann R.E., Kiss J.Z. (2011). An endogenous growth pattern of roots is revealed in seedlings grown in microgravity. Astrobiology.

[41] Karahara I., Suto T., Yamaguchi T., Yashiro U., Tamaoki D., Okamoto E., Yano S., Tanigaki F., Shimazu T., Kasahara H. (2020). Vegetative and reproductive growth of Arabidopsis under microgravity conditions in space. J. Plant Res..

[42] Hoson T., Soga K. (2003). New aspects of gravity responses in plant cells. Int. Rev. Cytol..

[43] Matia I., Gonzalez-Camacho F., Herranz R., Kiss J.Z., Gasset G., van Loon J.J., Marco R., Medina F.J. (2010). Plant cell proliferation and growth are altered by microgravity conditions in spaceflight. J. Plant Physiol..

[44] Hoson T., Soga K., Mori R., Saiki M., Nakamura Y., Wakabayashi K., Kamisaka S. (2002). Stimulation of elongation growth and cell wall loosening in rice coleoptiles under microgravity conditions in space. Plant Cell Physiol..

[45] Soga K., Wakabayashi K., Kamisaka S., Hoson T. (2002). Stimulation of elongation growth and xyloglucan breakdown in *Arabidopsis* hypocotyls under microgravity conditions in space. Planta.

[46] Soga K., Yamazaki C., Kamada M., Tanigawa N., Kasahara H., Yano S., Kojo K.H., Kutsuna N., Kato T., Hashimoto T. (2018). Modification of growth anisotropy and cortical microtubule dynamics in Arabidopsis hypocotyls grown under microgravity conditions in space. Physiol. Plant..

[47] Wakabayashi K., Soga K., Hoson T., Kotake T., Kojima M., Sakakibara H., Yamazaki T., Higashibata A., Ishioka N., Shimazu T. (2017). Persistence of plant hormone levels in rice shoots grown under microgravity conditions in space: Its relationship to maintenance of shoot growth. Physiol. Plant..

[48] Zhang Y., Wang L.H., Xie J.Y., Zheng H.Q. (2015). Differential protein expression profiling of Arabidopsis thaliana callus under microgravity on board the Chinese SZ-8 spacecraft. Planta.

[49] Hilaire E., Peterson B.V., Guikema J.A., Brown C.S. (1996). Clinorotation affects morphology and ethylene production in soybean seedlings. Plant Cell Physiol..

[50] Land E.S., Sheppard J., Doherty C.J., Perera I.Y. (2024). Conserved plant transcriptional responses to microgravity from two consecutive spaceflight experiments. Front. Plant Sci..

[51] Yamazaki C., Yamazaki T., Kojima M., Takebayashi Y., Sakakibara H., Uheda E., Oka M., Kamada M., Shimazu T., Kasahara H. (2023). Comprehensive analyses of plant hormones in etiolated pea and maize seedlings grown under microgravity conditions in space: Relevance to the International Space Station experiment “Auxin Transport”. Life Sci. Space Res..

[52] Barker R., Kruse C.P.S., Johnson C., Saravia-Butler A., Fogle H., Chang H.S., Trane R.M., Kinscherf N., Villacampa A., Manzano A. (2023). Meta-analysis of the space flight and microgravity response of the *Arabidopsis* plant transcriptome. NPJ Microgravity.

[53] Hausmann N., Fengler S., Hennig A., Franz-Wachtel M., Hampp R., Neef M. (2013). Cytosolic calcium, hydrogen peroxide and related gene expression and protein modulation in *Arabidopsis thaliana* cell cultures respond immediately to altered gravitation: Parabolic flight data. Plant Biol..

[54] Barjaktarović Ž., Nordheim A., Lamkemeyer T., Fladerer C., Madlung J., Hampp R. (2007). Time-course of changes in amounts of specific proteins upon exposure to hyper-g, 2-D clinorotation, and 3-D random positioning of *Arabidopsis* cell cultures. J. Exp. Bot..

[55] Olanrewaju G.O., Haveman N.J., Naldrett M.J., Paul A.L., Ferl R.J., Wyatt S.E. (2023). Integrative transcriptomics and proteomics profiling of *Arabidopsis thaliana* elucidates novel mechanisms underlying spaceflight adaptation. Front. Plant Sci..

[56] De Francesco S., Le Disquet I., Pereda-Loth V., Tisseyre L., De Pascale S., Amitrano C., Carnero Diaz E., De Micco V. (2024). Combined effects of microgravity and chronic low-dose gamma radiation on *Brassica rapa* microgreens. Plants.

[57] Mazars C., Brière C., Grat S., Pichereaux C., Rossignol M., Pereda-Loth V., Eche B., Boucheron-Dubuisson E., Le Disquet I., Medina F.J. (2014). Microsome-associated proteome modifications of Arabidopsis seedlings grown on board the International Space Station reveal the possible effect on plants of space stresses other than microgravity. Plant Signal. Behav..

[58] Kamada M., Oka M., Miyamoto K., Uheda E., Yamazaki C., Shimazu T., Sano H., Kasahara H., Suzuki T., Higashibata A. (2020). Microarray profile of gene expression in etiolated *Pisum sativum* seedlings grown under microgravity conditions in space: Relevance to the International Space Station experiment “Auxin Transport”. Life Sci. Space Res..

[59] Ha H.J., Subburaj S., Kim Y.S., Kim J.B., Kang S.Y., Lee G.J. (2020). Molecular characterization and identification of *calnexin 1* as a radiation biomarker from *Tradescantia* BNL4430. Plants.

[60] Valdez B.C., Li Y., Murray D., Liu Y., Nieto Y., Bashir Q., Qazilbash M.H., Andersson B.S. (2020). Panobinostat and venetoclax enhance the cytotoxicity of gemcitabine, busulfan, and melphalan in multiple myeloma cells. Exp. Hematol..

[61] Komatsu S., Hamada K., Furuya T., Nishiuchi T., Tani M. (2023). Membrane Proteomics to Understand Enhancement Effects of Millimeter-Wave Irradiation on Wheat Root under Flooding Stress. Int. J. Mol. Sci..

[62] Sarwat M., Naqvi A.R. (2013). Heterologous expression of rice calnexin (OsCNX) confers drought tolerance in *Nicotiana tabacum*. Mol. Biol. Rep..

[63] Chevet E., Smirle J., Cameron P.H., Thomas D.Y., Bergeron J.J. (2010). Calnexin phosphorylation: Linking cytoplasmic signalling to endoplasmic reticulum lumenal functions. Semin. Cell Dev. Biol..

[64] Vizcaíno J.A., Côté R.G., Csordas A., Dianes J.A., Fabregat A., Foster J.M., Griss J., Alpi E., Birim M., Contell J. (2013). The PRoteomics IDEntifications (PRIDE) database and associated tools: Status in 2013. Nucleic Acids Res..

[65] Okuda S., Watanabe Y., Moriya Y., Kawano S., Yamamoto T., Matsumoto M., Takami T., Kobayashi D., Araki N., Yoshizawa A.C. (2017). jPOSTTrepo: An international standard data repository for proteomes. Nucleic Acids Res..

