# Supplementary material for: The Growth of Soybean (Glycine max) Under Salt Stress Is Modulated in Simulated Microgravity Conditions
Source: Cells. 2025 Apr 3;14(7):541. doi: 10.3390/cells14070541 (PMC11988762; doi:10.3390/cells14070541)
Supplement: Supplementary file 1 [file cells-14-00541-s001.zip › Figures S1-S7.pdf]

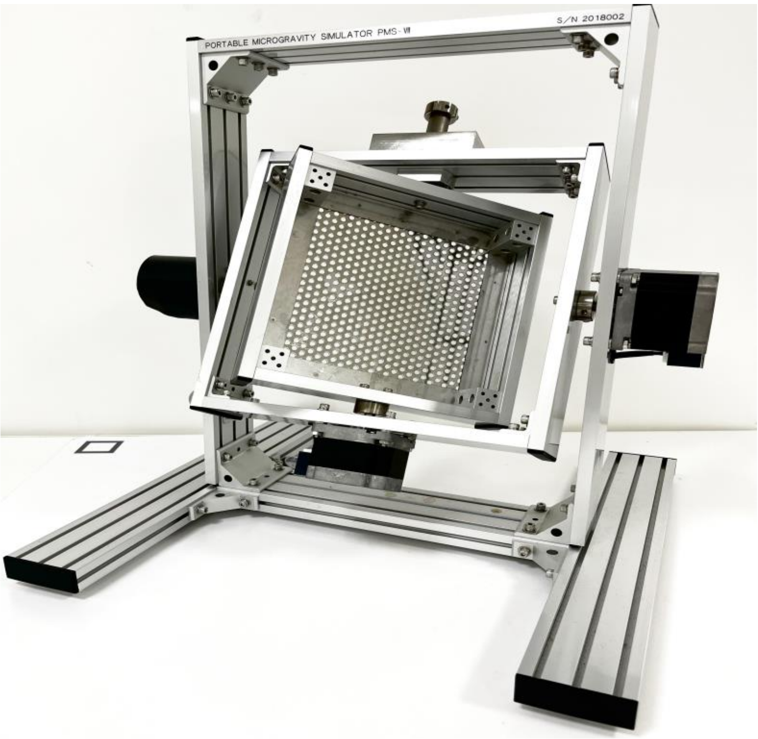

Figure S1. Device for simulated microgravity condition.

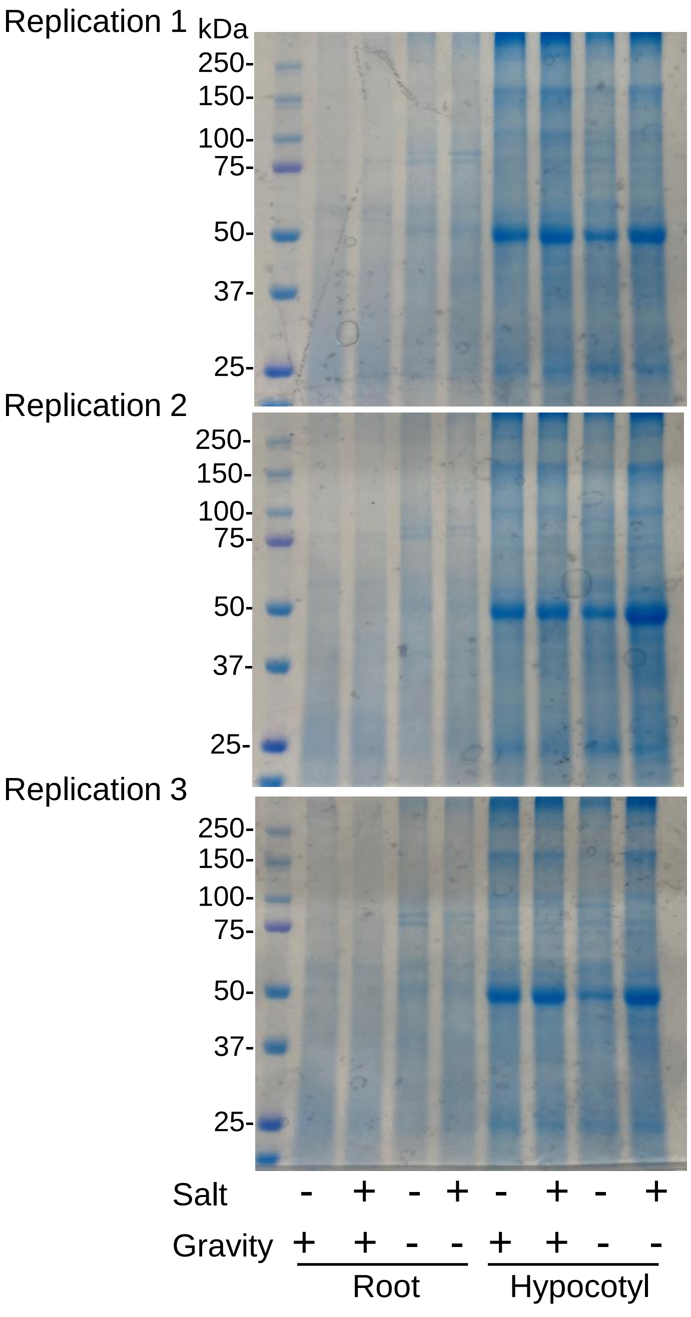

Figure S2: Coomassie-brilliant blue staining pattern of proteins used for immunoblot analysis. Experiments were performed with biologically triplicates for each treatments. Quantified proteins (10 µg) from leaf and root were separated by electrophoresis on a 10% SDS-polyacrylamide. Coomassie brilliant blue staining was used as loading control.

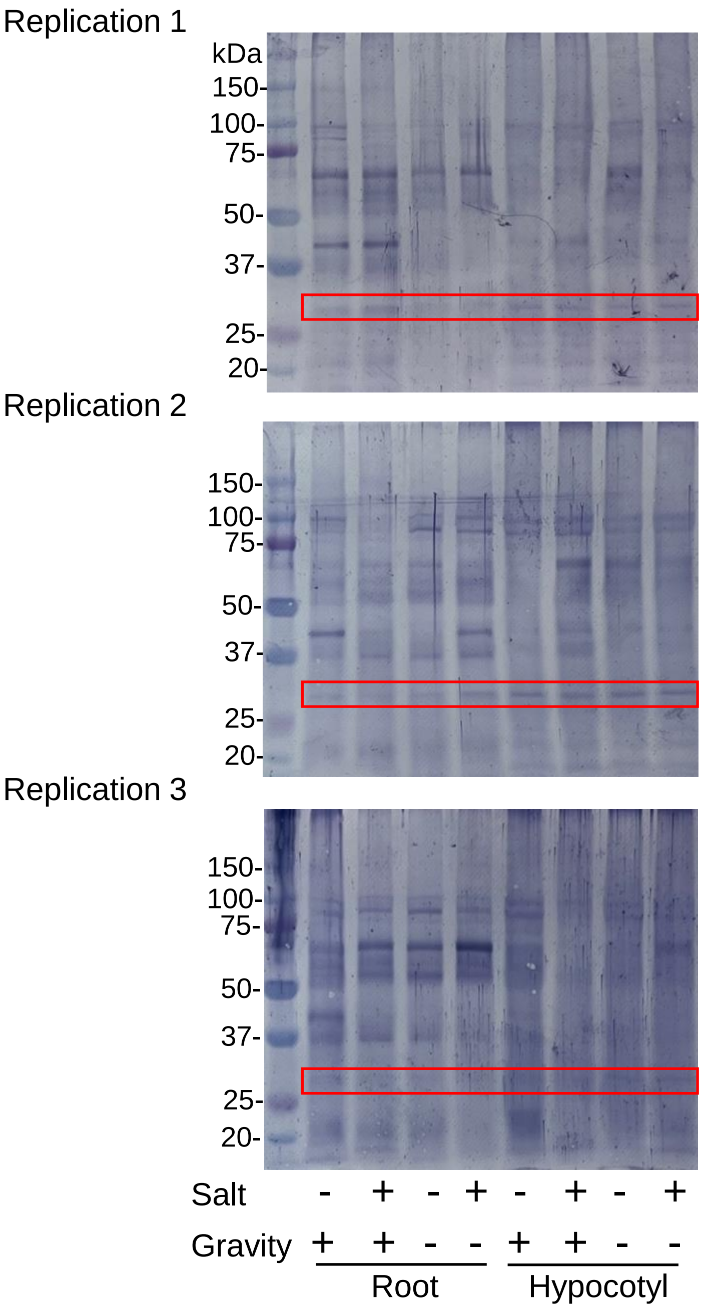

Figure S3. Blots of the entire membrane with anti-osmotin antibodies, which is used in Figure 7A.

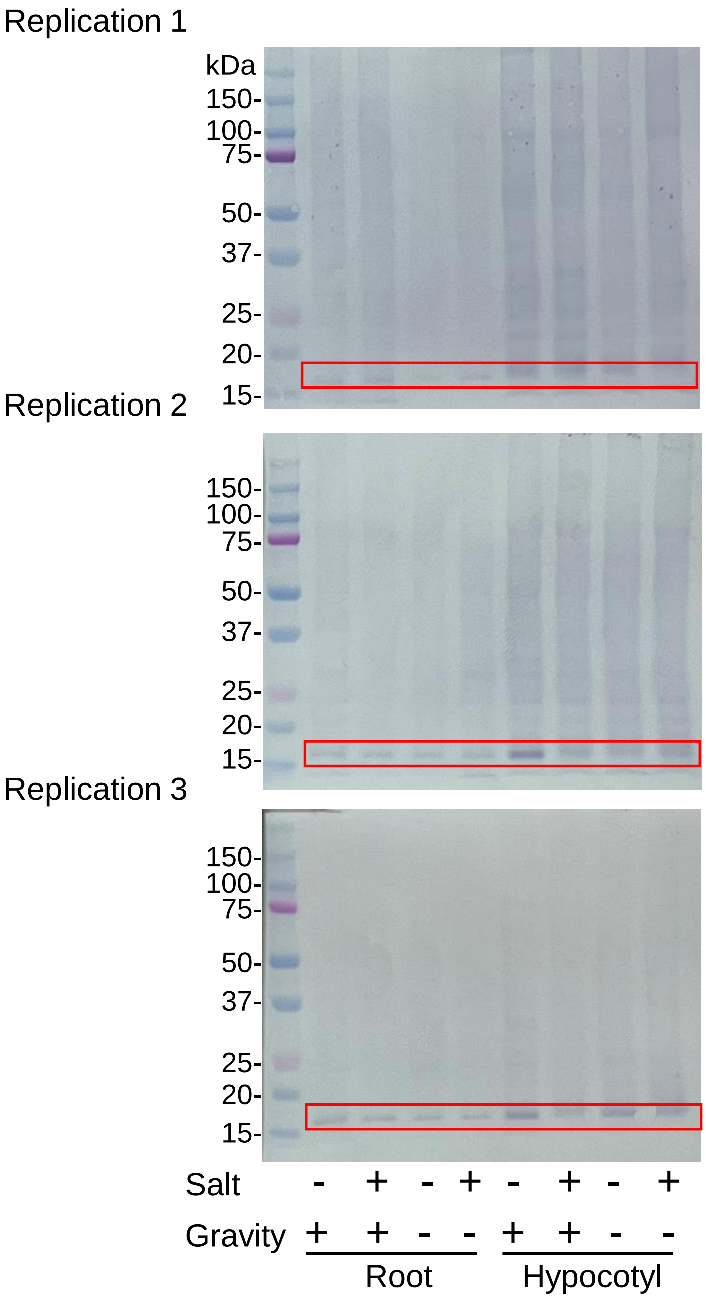

Figure S4. Blots of the entire membrane with anti-SOD antibodies, which is used in Figure 7B.

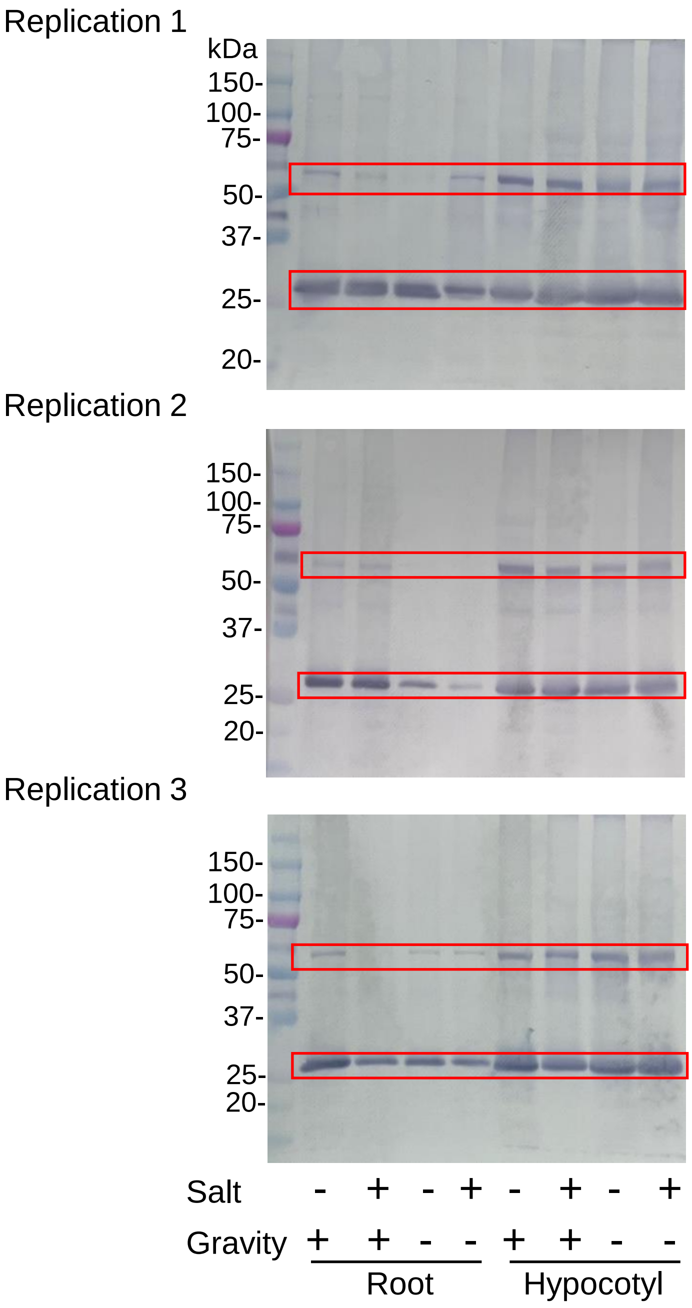

Figure S5. Blots of the entire membrane with anti-APX antibodies, which is used in Figure 7C and D..

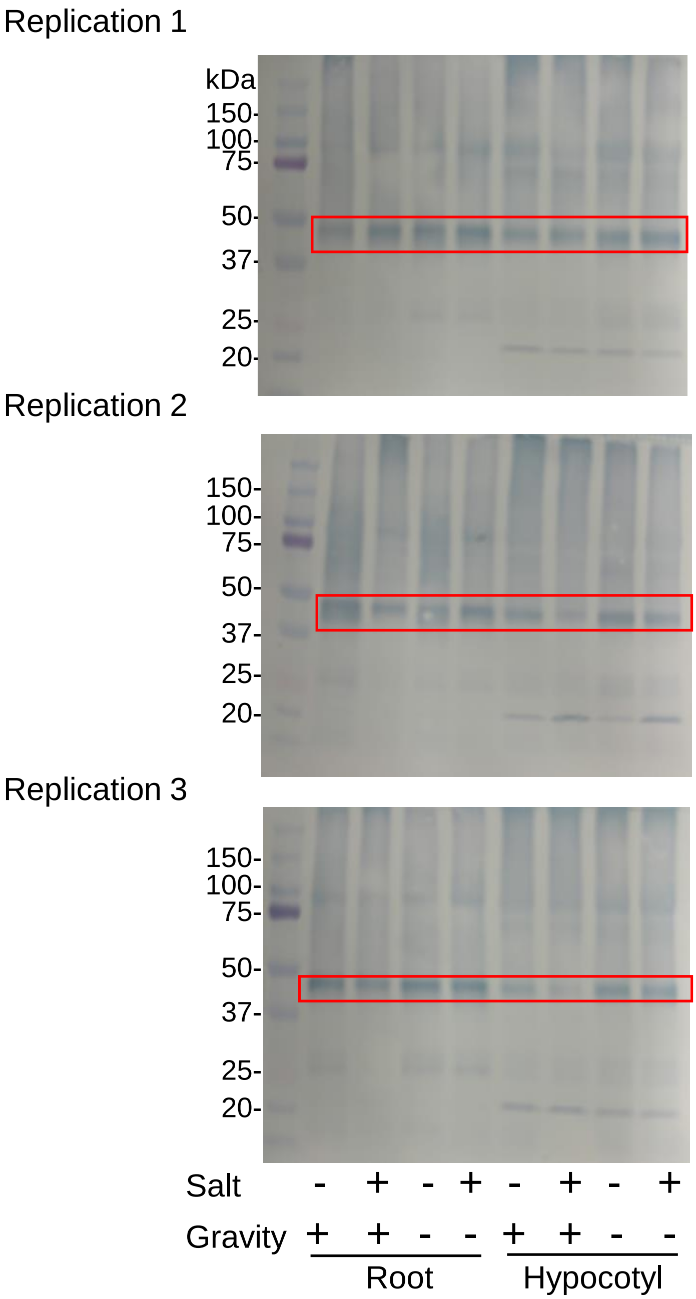

Figure S6. Blots of the entire membrane with anti-aquaporin antibodies, which is used in Figure 8A..

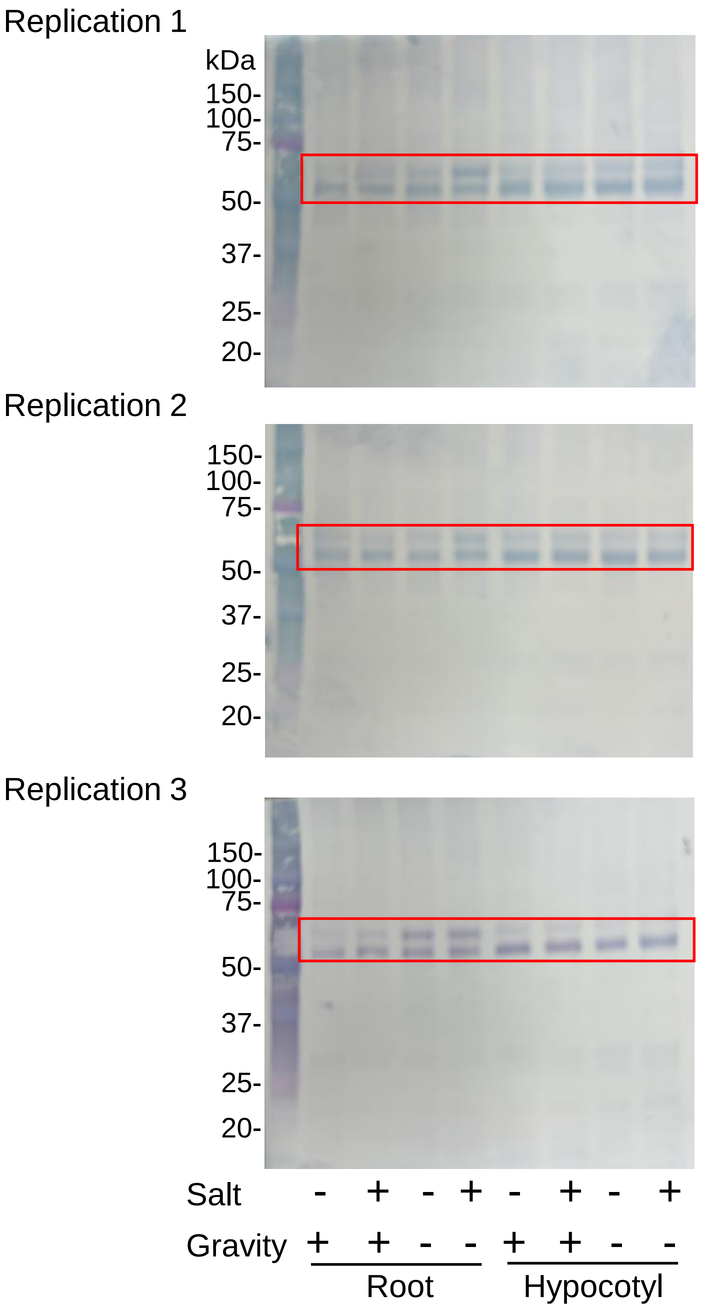

Figure S7. Blots of the entire membrane with anti-calreticulin antibodies, which is used in Figure 8B..
