# Supplementary material for: The Growth of Soybean (Glycine max) Under Salt Stress Is Modulated in Simulated Microgravity Conditions
Source: Cells. 2025 Apr 3;14(7):541. doi: 10.3390/cells14070541 (PMC11988762; doi:10.3390/cells14070541)
Supplement: Supplementary file 1 [file cells-14-00541-s001.zip › Table S1.pdf]

Table S1. Protein identification using nanoLC-MS/MS, MS data analysis, and differential analysis of proteins using MS data

Title	Methods
Protein identification using nanoLC-MS/MS	The peptides were loaded onto the LC system equipped with a trap column (Acclaim PepMap 100 C18 LC column; 3 µm, 75 µm ID x 20 mm; Thermo Fisher Scientific), equilibrated with 0.1% formic acid, and eluted with a linear acetonitrile gradient (0-35%) in 0.1% formic acid at a flow rate of 300 nL min^{-1}. The eluted peptides were loaded and separated on the column (EASY-Spray C18 LC column; 3 µm, 75 µm ID x 150 mm; Thermo Fisher Scientific) with a spray voltage of 2 kV (Ion Transfer Tube temperature: 275°C). The peptide ions were detected using MS in the data-dependent acquisition mode with the Xcalibur software (version 4.0; Thermo Fisher Scientific). Full-scan mass spectra were acquired in the MS over 375-1500 m/z with a resolution of 120000. The most intense precursor ions were selected for collision-induced fragmentation in the linear ion trap at a normalized collision energy of 35%. Dynamic exclusion was employed within 60 sec to prevent repetitive selection of peptides.
MS data analysis	Both algorithms included spectrum files RC, spectrum selector, MASCOT, SEQUEST HT search nodes, percolator, ptmRS, and minor feature detector nodes. The oxidation of methionine and carbamidomethylation of cysteine were set as a variable modification and a fixed modification, respectively. Mass tolerances in MS and MS/MS were set at 10 ppm and 0.6 Da, respectively. Trypsin was specified as protease and a maximum of 2 missed cleavages was allowed. Target-decoy database searches were used for the calculation of false discovery rate, which was calibrated at 1% for peptide identification.
Differential analysis of proteins using MS data	The abundances of proteins and peptides were transferred into log2 scale. Three biological replicates of each sample were grouped and a minimum of three valid values were required in one group. The normalization of the abundances was performed to subtract the median of each sample. Missing values were imputed based on a normal distribution (width=0.3, down-shift=1.8). Significance was assessed using Student's *t*-test analysis.
